# Antenatal corticosteroids for women at risk of imminent preterm birth in low-resource countries: the case for equipoise and the need for efficacy trials

**DOI:** 10.1136/bmjgh-2017-000398

**Published:** 2017-08-30

**Authors:** Joshua P Vogel, Olufemi T Oladapo, Cynthia Pileggi-Castro, Ebunoluwa A Adejuyigbe, Fernando Althabe, Shabina Ariff, Adejumoke Idowu Ayede, Abdullah H Baqui, Anthony Costello, Davy M Chikamata, Caroline Crowther, Bukola Fawole, Luz Gibbons, Alan H Jobe, Monica Lulu Kapasa, John Kinuthia, Alka Kriplani, Oluwafemi Kuti, James Neilson, Janna Patterson, Gilda Piaggio, Rahat Qureshi, Zahida Qureshi, Mari Jeeva Sankar, Jeffrey S A Stringer, Marleen Temmerman, Khalid Yunis, Rajiv Bahl, A Metin Gülmezoglu

**Affiliations:** 1 UNDP/UNFPA/UNICEF/WHO/World Bank Special Programme of Research, Development and Research Training in Human Reproduction (HRP), Department of Reproductive Health and Research, World Health Organization, Geneva, Switzerland; 2 Department of Maternal Newborn, Child and Adolescent Health, World Health Organization, Geneva, Switzerland; 3 Department of Paediatrics and Child Health, Obafemi Awolowo University, Ife, Nigeria; 4 Department of Mother and Child Health Research for Clinical Effectiveness and Health Policy (IECS), Buenos Aires, Argentina; 5 Department of Pediatrics & Child Health, Aga Khan University, Karachi, Pakistan; 6 Department of Paediatrics, College of Medicine, University of Ibadan, Ibadan, Nigeria; 7 International Center for Maternal and Newborn Health, Johns Hopkins Bloomberg School of Public Health, Baltimore, Maryland, USA; 8 Ministry of Community Development, Mother & Child Health, Lusaka, Zambia; 9 Liggins Institute, The University of Auckland, Auckland, New Zealand; 10 Department of Obstetrics & Gynaecology, College of Medicine, University of Ibadan, Ibadan, Nigeria; 11 Department of Pediatrics, Cincinnati Childrens Hospital, Cincinnati, Ohio, USA; 12 Department of Paediatrics, University Teaching Hospital, Lusaka, Zambia; 13 Department of Research & Programs, Kenyatta National Hospital, Nairobi, Kenya; 14 All India Institute of Medical Sciences, New Delhi, India; 15 Department of Obstetrics, Gynaecology and Perinatology, College of Health Sciences, Obafemi Awolowo University, Ife, Nigeria; 16 Department of Women's and Children's Health, The University of Liverpool, Liverpool, UK; 17 Maternal, Newborn, and Child Health, Bill and Melinda Gates Foundation, Geneva, Switzerland; 18 Department of Medical Statistics, London School of Hygiene and Tropical Medicine, London, UK; 19 Department of Obstetrics and Gynecology, Aga Khan University, Karachi, Pakistan; 20 Department of Obstetrics and Gynaecology, School of Medicine, University of Nairobi, Nairobi, Kenya; 21 Department of Pediatrics, WHO Collaborating Centre for Training and Research in Newborn Care, All India Institute of Medical Sciences (AIIMS), New Delhi, India; 22 University of North Carolina, School of Medicine, Chapel Hill, North Carolina, USA; 23 National Collaborative Perinatal Neonatal Network, American University of Beirut, Beirut, Lebanon

**Keywords:** preterm birth, antenatal corticosteroids, neonatal mortality

## Abstract

The scientific basis for antenatal corticosteroids (ACS) for women at risk of preterm birth has rapidly changed in recent years. Two landmark trials—the Antenatal Corticosteroid Trial and the Antenatal Late Preterm Steroids Trial—have challenged the long-held assumptions on the comparative health benefits and harms regarding the use of ACS for preterm birth across all levels of care and contexts, including resource-limited settings. Researchers, clinicians, programme managers, policymakers and donors working in low-income and middle-income countries now face challenging questions of whether, where and how ACS can be used to optimise outcomes for both women and preterm newborns.

In this article, we briefly present an appraisal of the current evidence around ACS, how these findings informed WHO’s current recommendations on ACS use, and the knowledge gaps that have emerged in the light of new trial evidence. Critical considerations in the generalisability of the available evidence demonstrate that a true state of clinical equipoise exists for this treatment option in low-resource settings. An expert group convened by WHO concluded that there is a clear need for more efficacy trials of ACS in these settings to inform clinical practice.

**Key messages**

**What is already known about this topic?**

- Antenatal corticosteroids (ACS) have been considered the gold standard treatment for reducing preterm-associated neonatal morbidity and mortality for decades. However, the unexpected findings of the recently published Antenatal Corticosteroids Trial in low-income and middle-income countries (LMICs) have challenged long-held assumptions on the comparative health benefits and harms of ACS use in LMICs across of all levels of care.
- In 2015, WHO recommended ACS use up to 34 weeks’ gestation with strict treatment criteria to guide its use, in acknowledgement of unanswered questions about safety and efficacy in hospitals in low-resource countries.

**New information**

- A reappraisal of the hallmark Cochrane review evidence identified several limitations that undermine generalisability to lower-income countries. Furthermore, the recent Antenatal Late Preterm Steroids trial conducted in tertiary centres in the USA reported some benefits for late preterm newborns. Questions still remain regarding the applicability of this new evidence to hospitals in low-resource countries.
- WHO convened an expert group that reviewed the available evidence—the group agreed that efficacy trials on ACS use for preterm birth in hospitals in low-resource countries are justified, to guide clinicians and policymakers on whether, how and where ACS can be used safely and effectively in these settings.

## The global burden and risks of preterm birth

Preterm birth is defined as live births occurring before 37 completed weeks of gestation.^1^ An estimated 14.9 million neonates were born preterm in 2010, accounting for 11.1% of live births worldwide.^2^ The majority of all preterm births occur in the late preterm period (34 to <37 weeks)—for example, in the USA more than 70% of preterm births in 2014 were born in the late preterm period.^3^ It is estimated that more than 60% of the world’s preterm births occur in sub-Saharan African and South Asian countries.^2^

Prematurity can be a lethal condition, particularly for those newborns born at earlier gestational ages. Complications of preterm birth are the leading cause of death in children under 5 years of age globally, accounting for 1.06 million deaths (uncertainty range 0.935 to 1.179 million) of the 5.9 million deaths estimated to have occurred in 2015.^4^ Those preterm neonates who survive are at increased risk of a wide range of respiratory, infectious, metabolic and neurological morbidities. Preterm infants experience higher rates of respiratory distress syndrome, bronchopulmonary dysplasia, necrotising enterocolitis, kernicterus, hypoglycaemia, periventricular leucomalacia, seizures, intraventricular haemorrhage, cerebral palsy, infections, feeding difficulties, hypoxic ischaemic encephalopathy, retinopathy of prematurity, as well as visual and hearing loss.^5–16^ Preterm birth and its sequelae can have significant negative psychosocial and financial impacts on families of preterm newborns.^17–20^

While the risks of mortality and morbidity affecting preterm newborns are considerably more frequent at lower gestational ages,^11^ late preterm infants (sometimes called ‘near-term’) still experience significantly higher risks compared with babies born at term.^21^ A systematic review of more than 29 million infants (mostly in high-income countries) found that, compared with term birth, late preterm birth was associated with increased 28-day mortality (Risk Ratio (RR) 5.9, 95% CI 5.0 to 6.9) and 1-year mortality (RR 3.7, 95% CI 2.9 to 4.6).^21^

## Antenatal corticosteroids

Antenatal corticosteroids (ACS) have long been regarded as a cornerstone intervention in mitigating the adverse effects of preterm birth. The Cochrane Collaboration logo is itself constructed from an early ACS meta-analysis.^22^ The first randomised controlled trial of ACS (betamethasone) in humans to prevent respiratory distress syndrome was published in 1972.^23^ Since then, dozens more trials have been conducted, exploring neonatal risks and benefits when given to women at risk of preterm birth,^24^ the use of different dosing regimens^25^ and the use of ACS for preventing neonatal respiratory morbidity after elective caesarean section at term.^26^

In March 2017, the updated Cochrane systematic review on ACS for accelerating fetal lung maturation for women at risk of preterm birth was published, including 30 trials of 7774 women and 8158 infants.^27^ The findings are similar to earlier iterations, showing striking reductions in neonatal mortality and several morbidity outcomes (box 1). This analysis has contributed to the widespread (and often overly liberal) use of ACS for women at risk of preterm birth, including in low-income and middle-income countries (LMICs).^28^

Box 1 **Neonatal outcomes of antenatal corticosteroids for accelerating fetal lung maturation from updated Cochrane review^27^**

- 32% reduction in neonatal deaths (Risk Ratio (RR) 0.69, 95% CI 0.59 to 0.81; 22 studies of 7188 participants)
- 35% reduction in respiratory distress syndrome (RDS) (average RR 0.66, 95% CI 0.56 to 0.77; 28 studies of 7764 participants)
- 45% reduction in moderate and severe RDS (average RR 0.59, 95% CI 0.38 to 0.91; 6 studies of 1686 participants)
- 46% reduction in intraventricular haemorrhage (average RR 0.55, 95% CI 0.40 to 0.76; 16 studies of 6093 participants)
- 54% reduction in necrotising enterocolitis (RR 0.50, 95% CI 0.32 to 0.78; 10 studies of 4702 participants)
- 43% reduction in infant systemic infection in the first 48 hours of life (RR 0.60, 95% CI 0.41 to 0.88; 8 studies of 1753 participants)

However, there are several critical limitations in the Cochrane review evidence base that complicate their application to many hospitals in LMICs, as discussed below.

## A reappraisal of the Cochrane review evidence

### Trial settings

The 30 trials were conducted in higher-level hospital settings, in high-income (20 trials) and upper middle-income (nine trials) countries, except one trial that was conducted in Tunisia (a lower middle income country).^29^ It seems reasonable to assume that the level of maternal and newborn care provided reflected the best available at the time the studies were conducted, including the accuracy of gestational age estimation for recruited women. Comparatively, no placebo-controlled efficacy trials of ACS have been conducted in low-income countries, where the rates of maternal and newborn mortality and morbidity are higher, and the level of health and human resources available to manage pregnant women and preterm infants substantially lower. Despite this, ACS are routinely used in facilities in many lower-income countries.^30^

### Age of the trials and risk of bias

The Antenatal Late Preterm Steroid (ALPS) Trial published in 2016^31^ (discussed further below) is the largest trial in this meta-analysis. Among the other 29 trials, three-quarters of participants were randomised more than 20 years ago. While the age of a trial itself is not necessarily an indication of poor quality, reports of older trials often contain no or insufficient information to fully assess their risk of bias. Importantly, the context of maternal and newborn care has changed substantially since those trials were conducted—interventions such as oxygen therapy, continuous positive airway pressure (CPAP), thermal care, nutritional support, mechanical ventilation and surfactant are more widely used now than in past decades. Given these improvements, the anticipated benefits of ACS may therefore not be as large as expected. It is noteworthy that infant mortality due to respiratory distress syndrome in the USA has decreased significantly since the 1970s,^32^ with large reductions achieved prior to the widespread use of ACS.^33^

### Heterogeneity in participants between trials

There is considerable heterogeneity in the eligibility criteria used in these trials; table 1 gives the different gestational age ranges used. Trials have included or excluded women with certain obstetric characteristics—women in spontaneous preterm labour, women with premature prelabour rupture of membranes, women with planned preterm birth and women with high-risk obstetric conditions (such as multiple pregnancies, diabetes, infection and hypertensive disorders). The pooling of data when trials are so diverse may be inappropriate. Furthermore, the preterm birth rate after randomisation among women recruited to these trials was generally very high. At least 11 trials had preterm birth rates at or near 100%, suggesting that these trials recruited women who had a very high likelihood of delivering preterm. It also raises the possibility that some infants exposed to ACS were ultimately born at term, but were not captured or included for analysis.

**Table 1:** Gestational age ranges used in eligibility criteria for antenatal corticosteroids administration, reproduced with permission from updated Cochrane review^24^

Study	Gestational age range
Amorim 1999	28 weeks 0 days to 34 weeks 6 days
Attawattanakul 2015	34 weeks 0 days to 36 weeks 6 days
Balci 2010	34 weeks 0 days to 36 weeks 6 days
Block 1977	Up to 36 weeks 6 days*
Cararach 1991	28 weeks 0 days to 30 weeks 6 days
Carlan 1991	28 weeks 0 days to 30 weeks 6 days
Collaborative 1981	26 weeks 0 days to 37 weeks 0 days
Dexiprom 1999	28 weeks 0 days to 34 weeks 6 days (or estimated fetal weight 1000–2000 g)
Doran 1980	24 weeks 0 days to 34 weeks 6 days
Fekih 2002	26 weeks 0 days to 34 weeks 6 weeks
Gamsu 1989	Up to 34 weeks 6 days*
Garite 1992	24 weeks 0 days to 27 weeks 6 days
Goodner 1979	Up to 33 weeks 6 days*
Gyamfi-Bannerman 2016	34 weeks 0 days to 36 weeks 6 days
Kari 1994	24 weeks 0 days to 31 weeks 6 days
Lewis 1996	24 weeks 0 days to 34 weeks 6 days
Liggins 1972	24 weeks 0 days to 36 weeks 6 days
Lopez 1989	27 weeks 0 days to 35 weeks 0 days
Mansouri 2010	25 weeks 0 days to 36 weeks 6 days
Morales 1989	26 weeks 0 days to 34 weeks 6 days
Nelson 1985	28 weeks 0 days to 34 weeks 6 days
Parsons 1988	25 weeks 0 days to 32 weeks 6 days
Porto 2011	34 weeks 0 days to 36 weeks 6 days
Qublan 2001	27 weeks 0 days to 34 weeks 6 days
Schutte 1980	26 weeks 0 days to 32 weeks 6 days
Shanks 2010	34 weeks 0 days to 36 weeks 6 days
Silver 1996	24 weeks 0 days to 29 weeks 6 days
Taeusch 1979	Up to 33 weeks 6 days*
Teramo 1980	28 weeks 0 days to 35 weeks 6 days

*Lower gestational age limit not specified.

### Measurement of neonatal mortality

Twenty-two trials reported on neonatal death; however, none were independently powered to detect a difference in this outcome. All were facility based, and in general did not specify what definition of neonatal death was used, nor how the follow-up of newborns to ascertain vital status was conducted. Importantly, several trials had excluded women postrandomisation, some of which may have directly impacted on detection of neonatal mortality. In total, this meta-analysis included 551 deaths in 6729 newborns (a mortality rate of 8.2% overall). In low-resource countries, newborn mortality rates in preterm newborns can be two to three times higher.

Only seven trials had ≥200 newborns each, accounting for 78% of the total sample size for this outcome. The ALPS Trial, by far the largest trial for this outcome, reported two deaths in the intervention arm and zero in the control arm (a non-significant difference). None of the six largest trials independently reported a reduction in the risk of neonatal death. The seventh (Amorim *et al*) randomised 218 pregnant women and reported a 50% risk reduction in neonatal death (RR 0.50, 95% CI 0.28 to 0.89); however, this trial included only women with severe pre-eclampsia.^34^ The remaining 15 trials were all small (<200 newborns each); three trials had <100 newborns. Only four small trials reported independent reductions in the risk of newborn mortality, with effect sizes exceeding 50%.^29 35–37^ The impact of a large number of small trials on the summary estimate is of concern. The funnel plot for this outcome does not indicate an obvious publication bias, but this does not rule out the possibility.^38^

## The Antenatal Corticosteroids Trial

In 2015, Althabe and colleagues published findings from The Antenatal Corticosteroids Trial (ACT). ACT was a community-based, cluster-randomised trial conducted in six LMICs: Argentina, Guatemala, India, Kenya, Pakistan and Zambia.^39^ The trial aimed to evaluate the feasibility, effectiveness and safety of a multifaceted intervention designed to increase the use of ACS at all levels of healthcare. The intervention included ACS commodity procurement, as well as training and tools for health providers to recognise at-risk women, estimate gestational age and administer dexamethasone. The primary outcome was neonatal death at 28 completed days among liveborn neonates at less than fifth percentile for birth weight (as a proxy for preterm births, because of inadequate gestational age information).

ACT included 101 clusters in six countries, capturing nearly 100 000 live births. The use of ACS increased in the intervention arm for women with a less-than-fifth-percentile infant (45% vs 10%), but also for all women, regardless of her baby’s birth weight (12% vs 2%). Only 16% of the women who were given ACS ultimately gave birth to a less-than-fifth-percentile newborn, highlighting substantial overdiagnosis of imminent preterm birth and overtreatment with ACS. Among the less-than-fifth-percentile newborns, ACS use had no effect on neonatal deaths (RR 0.96, 95% CI 0.87 to 1.06). However, among all births, there were *increased* risks of neonatal mortality (RR 1.12, 1.02–1.22) and stillbirth (RR 1.11, 1.02–1.22)—a very unexpected and concerning finding. The authors reported that the increased mortality was seemingly driven by increased mortality in infants above the 25th percentile for birth weight. Furthermore, the intervention was associated with an increased OR of suspected maternal infection in women with less-than-fifth-percentile babies (OR 1.67, 95% CI 1.33 to 2.09), and all women (OR 1.45, 95% CI 1.33 to 1.58).

While this complex intervention successfully increased the use of ACS more than fourfold in low-resource settings, the overall picture was alarming—lack of benefit in the less-than-fifth-percentile newborns, evidence of increased perinatal mortality in larger newborns and the increase in suspected maternal infections. While the exact causes are not known, the study group hypothesise that maternal and newborn infections may play a role.^40^

## WHO recommendations on use of ACS (2015)

How then should these two contrasting bodies of evidence—30 randomized controlled trials largely from high-resourced hospitals in higher-income countries, and a large implementation trial in six LMICs—be balanced? The WHO recommendations on interventions to improve preterm birth outcomes were published in 2015, for which an updated search and meta-analysis was conducted.^41^ WHO recommends ACS for women at risk of preterm birth from 24 weeks to 34 weeks gestation in settings where certain criteria are met (box 2).^42^ These consensus-based treatment criteria were intended to address the issues regarding safety of ACS in resource-limited settings. The recommendation remarks specify that ACS should not be routinely administered in situations where the gestational age cannot be confirmed (particularly when gestational age is suspected to be more than 34 weeks), as the risk of neonatal harm may outweigh the benefits.

Box 2 **Antenatal corticosteroids treatment criteria in the WHO recommendations to improve preterm birth outcomes^42^**

- Gestational age assessment can be accurately undertaken
- Preterm birth is considered imminent
- There is no clinical evidence of maternal infection
- Adequate childbirth care is available (including the capacity to recognise and safely manage preterm labour and birth)
- The preterm newborn can receive adequate care if needed (including resuscitation, thermal care, feeding support, infection treatment and safe oxygen use)

The ACT Trial highlighted that scaling up ACS in LMICs without more definitive evidence on benefits and harms could prove detrimental to both mothers and newborns. Given the uncertainties inherent in generalising the current evidence base to facilities in low-resource settings, and the risk of harm at higher gestational ages, the WHO guideline panel recommended further research to inform ACS use and improve generalisability to lower-resource settings.^41^

## The Antenatal Late Preterm Steroids Trial

The findings of the Antenatal Late Preterm Steroids (ALPS) Trial were published in early 2016 (after the release of the WHO recommendations).^31^ This was a multicentre, randomised trial in tertiary care centres in the USA that recruited women with a singleton pregnancy, at high risk for preterm birth and at 34 weeks 0 days to 36 weeks 5 days of gestation. Participants were randomised to receive up to two injections of betamethasone or matching placebo, 24 hours apart. The primary outcome was a management-based, composite severe adverse neonatal outcome relating to need for respiratory support. It was defined as one or more of the use of CPAP or high-flow nasal cannula for at least two consecutive hours, supplemental oxygen with a fraction of inspired oxygen of at least 0.30 for at least four continuous hours, extracorporeal membrane oxygenation or mechanical ventilation. Stillbirth and neonatal death within 72 hours were included as competing events.

The authors reported that the primary outcome was significantly reduced in the intervention arm, 11.6% vs 14.4% (RR 0.80; 95% CI 0.66 to 0.97). Other newborn secondary outcomes (including severe respiratory complications, transient tachypnoea of the newborn, surfactant use and bronchopulmonary dysplasia) were also significantly less frequent in the betamethasone group, although neonatal hypoglycaemia was more common in the betamethasone group (24.0% vs 15.0%; RR 1.60; 95% CI 1.37 to 1.87). There were no apparent differences in the incidence of chorioamnionitis, respiratory distress syndrome or neonatal sepsis.

The ALPS Trial findings were meta-analysed with similar trials in a recent systematic review by Saccone and Berghella, who explored the role of ACS in term or near-term fetuses.^26^ The review included six trials, comprising 5698 singleton pregnancies. The review authors concluded that ACS at ≥34 weeks’ gestation reduces neonatal respiratory morbidity, and that a single course of corticosteroids can be considered for women at risk of imminent late premature birth (34 weeks 0 days to 36 weeks 6 days) gestation, as well as for the subgroup of women undergoing planned caesarean delivery at ≥37 weeks’ gestation.

WHO does not currently recommend the use of ACS in the late preterm period, given the lack of evidence of benefit at the time the WHO recommendations were developed. While the findings of the ALPS Trial suggest that ACS in the late preterm period could reduce newborn respiratory morbidity (but not fetal or neonatal mortality) in high-resource settings, it is not certain that these findings—which relate largely to reducing the need for newborn care interventions available in high-level hospitals—can be replicated in hospitals in LMICs where considerably fewer health and human resources are available. Late preterm ACS use might confer a mortality benefit in low-resource settings, where rates of neonatal mortality in late preterm newborns are unacceptably high, but this is speculative.

## The case for equipoise, and the need for efficacy trials in low-resource settings

In November 2015, WHO convened a technical consultation of obstetricians, neonatologists and researchers in preterm birth to review and discuss the knowledge gaps around ACS use prior to 34 weeks. With the publication of the ALPS Trial in February 2016, an additional meeting of researchers and technical advisors was held in July 2016 to review the evidence around the late preterm period.

Based on the evidence appraisal above, it was agreed that there is a clear justification for further randomised controlled trials to evaluate the efficacy of ACS in facility settings in lower-income countries. While evidence suggests that there may be a role for ACS in preterm birth management in these settings, efficacy evidence from hospitals in low-resource countries that balances possible maternal, fetal and neonatal benefits and harms is required to guide clinical practice.

The group noted that if the conduct of such an efficacy trial is limited to women at imminent risk of early preterm birth (<34 weeks), future recommendations on ACS use would continue to be restricted to this gestational age limit. This can complicate ACS scale-up in most low-resource countries, where accurate gestational age assessment is often not available. The outstanding question of possible benefits and harms for mothers and late preterm babies will also remain unresolved.

If a separate, independently powered efficacy trial of ACS in the late preterm period showed benefit, the public health impact will be significant. Compared with early preterm births, neonatal mortality rates are lower in late preterm babies, but the prevalence is more than three times larger. Even modest benefits (in the absence of harms) would thus translate into substantive impacts on preterm-associated morbidity, mortality and healthcare utilisation. If neither benefits nor harms are demonstrated in the late preterm period, reliance on accurate gestational age assessment around 34 weeks will be less critical. The various scenarios are summarised in table 2.

**Table 2:** Programmatic implications of conducting two concurrent trials of ACS, considering possible scenarios of effects on newborn mortality outcome

		Early preterm period (ACTION-I Trial)		
		Shows reduction in newborn mortality	Shows no reduction in newborn mortality	Shows increase in newborn mortality
Late preterm period (ACTION-II Trial)	Shows reduction in newborn mortality	Use ACS 26 to 36 weeks	Unlikely to occur	Unlikely to occur
	Shows no reduction in newborn mortality	Use ACS at 26 to <34 weeks Gestational age threshold not critical for safety	Do not use ACS	Do not use ACS
	Shows increase in newborn mortality	Use ACS at 26 to <34 weeks GA threshold is critical for safety	Do not use ACS	Do not use ACS

ACS, antenatal corticosteroids; ACTION, Antenatal CorticosTeroids for Improving Outcomes in preterm Newborns.

This has led to the establishment of an international research collaboration, called the WHO Antenatal CorticosTeroids for Improving Outcomes in preterm Newborns (WHO ACTION) Trials. With support from the Bill and Melinda Gates Foundation, this collaboration will conduct two concurrent and independently powered, hospital-based, placebo-controlled efficacy trials of ACS (dexamethasone), which will recruit women presenting to participating hospitals at imminent risk of preterm birth. The ACTION-I trial will randomise eligible women from 26 weeks 0 days to 33 weeks 6 days, while the ACTION-II trial will randomise eligible women from 34 weeks 0 days to 36 weeks 0 days. The trials will be conducted in hospitals with a sufficient level of maternal and newborn care in Bangladesh, India, Kenya, Nigeria and Pakistan. These hospitals will be supported (where necessary) with additional equipment and training, in order to optimise gestational age dating, as well as care of preterm newborns. When concluded, these two trials will add more than 28 000 women to the Cochrane review meta-analysis on this question, providing the needed trial evidence on ACS use in low-resource countries.

## References

[1] WHO: recommended definitions, terminology and format for statistical tables related to the perinatal period and use of a new certificate for cause of perinatal deaths. Modifications recommended by FIGO as amended. Acta Obstet Gynecol Scand 19761977;56:247–53. 560099

[2] Blencowe H, Cousens S, Oestergaard MZ, et al National, regional, and worldwide estimates of preterm birth rates in the year 2010 with time trends since 1990 for selected countries: a systematic analysis and implications. Lancet 2012;379:2162–72. doi:10.1016/S0140-6736(12)60820-4 22682464 10.1016/S0140-6736(12)60820-4

[3] Hamilton BE, Martin JA, Osterman M, et al Births: final Data for 2014. 64: National vital statistics reports: from the Centers for Disease Control and Prevention, National Center for Health Statistics, National Vital Statistics System:12. 26727629

[4] Liu L, Oza S, Hogan D, et al Global, regional, and national causes of under-5 mortality in 2000-15: an updated systematic analysis with implications for the Sustainable Development Goals. Lancet 2016;388:3027–35. doi:10.1016/S0140-6736(16)31593-8 27839855 10.1016/S0140-6736(16)31593-8 PMC5161777

[5] Islam JY, Keller RL, Aschner JL, et al Understanding the short- and Long-Term Respiratory outcomes of prematurity and bronchopulmonary dysplasia. Am J Respir Crit Care Med 2015;192:134–56. doi:10.1164/rccm.201412-2142PP 26038806 10.1164/rccm.201412-2142PP PMC4532824

[6] Schnabl KL, Van Aerde JE, Thomson AB, et al Necrotizing enterocolitis: a multifactorial disease with no cure. World J Gastroenterol 2008;14:2142–61. doi:10.3748/wjg.14.2142 18407587 10.3748/wjg.14.2142 PMC2703838

[7] Wallenstein MB, Bhutani VK Jaundice and kernicterus in the moderately preterm infant. Clin Perinatol 2013;40:679–88. doi:10.1016/j.clp.2013.07.007 24182955 10.1016/j.clp.2013.07.007

[8] Kinney HC The near-term (late preterm) human brain and risk for periventricular leukomalacia: a review. Semin Perinatol 2006;30:81–8. doi:10.1053/j.semperi.2006.02.006 16731282 10.1053/j.semperi.2006.02.006

[9] Ramenghi LA Late preterm babies and the risk of neurological damage. Acta Biomed 2015;86(Suppl 1):36–40. 26135955

[10] Mwaniki MK, Atieno M, Lawn JE, et al Long-term neurodevelopmental outcomes after intrauterine and neonatal insults: a systematic review. Lancet 2012;379:445–52. doi:10.1016/S0140-6736(11)61577-8 22244654 10.1016/S0140-6736(11)61577-8 PMC3273721

[11] Saigal S, Doyle LW An overview of mortality and sequelae of preterm birth from infancy to adulthood. Lancet 2008;371:261–9. doi:10.1016/S0140-6736(08)60136-1 18207020 10.1016/S0140-6736(08)60136-1

[12] Araújo BF, Zatti H, Madi JM, et al Analysis of neonatal morbidity and mortality in late-preterm newborn infants. J Pediatr 2012;88:259–66. doi:doi:http://dx.doi.org/10.2223/JPED.2196 10.2223/jped.2196 22717575

[13] Platt MJ Outcomes in preterm infants. Public Health 2014;128:399–403. doi:10.1016/j.puhe.2014.03.010 24794180 10.1016/j.puhe.2014.03.010

[14] Blencowe H, Lawn JE, Vazquez T, et al Preterm-associated visual impairment and estimates of retinopathy of prematurity at regional and global levels for 2010. Pediatr Res 2013;74(Suppl 1):35–49. doi:10.1038/pr.2013.205 24366462 10.1038/pr.2013.205 PMC3873709

[15] van Dommelen P, Verkerk PH, van Straaten HL Hearing loss by week of gestation and birth weight in very preterm neonates. J Pediatr 2015;166:840–3. doi:10.1016/j.jpeds.2014.12.041 25661409 10.1016/j.jpeds.2014.12.041

[16] O'Connor AR, Wilson CM, Fielder AR Ophthalmological problems associated with preterm birth. Eye 2007;21:1254–60. doi:10.1038/sj.eye.6702838 17914427 10.1038/sj.eye.6702838

[17] Bérard A, Le Tiec M, De Vera MA Study of the costs and morbidities of late-preterm birth. Arch Dis Child Fetal Neonatal Ed 2012;97:F329–F334. doi:10.1136/fetalneonatal-2011-300969 22933090 10.1136/fetalneonatal-2011-300969

[18] Singer LT, Salvator A, Guo S, et al Maternal psychological distress and parenting stress after the birth of a very low-birth-weight infant. JAMA 1999;281:799–805. doi:10.1001/jama.281.9.799 10071000 10.1001/jama.281.9.799 PMC10189739

[19] Korvenranta E, Lehtonen L, Rautava L, et al Impact of very preterm birth on health care costs at five years of age. Pediatrics 2010;125:e1109–e1114. doi:10.1542/peds.2009-2882 20368320 10.1542/peds.2009-2882

[20] Petrou S, Abangma G, Johnson S, et al Costs and health utilities associated with extremely preterm birth: evidence from the EPICure study. Value Health 2009;12:1124–34. doi:10.1111/j.1524-4733.2009.00580.x 19659702 10.1111/j.1524-4733.2009.00580.x

[21] Teune MJ, Bakhuizen S, Gyamfi Bannerman C, et al A systematic review of severe morbidity in infants born late preterm. Am J Obstet Gynecol 2011;205:374.e1–374.e9. doi:10.1016/j.ajog.2011.07.015 21864824 10.1016/j.ajog.2011.07.015

[22] Chalmers I Iain Chalmers: should the Cochrane logo be accompanied by a health warning?*[Internet]*. 2016 http://www.evidentlycochrane.net/cochrane-logo-health-warning/ (accessed 19 Jan 2017).

[23] Liggins GC, Howie RN A controlled trial of antepartum glucocorticoid treatment for prevention of the respiratory distress syndrome in premature infants. Pediatrics 1972;50:515–25. 4561295

[24] Roberts D, Dalziel S Antenatal corticosteroids for accelerating fetal lung maturation for women at risk of preterm birth. Cochrane Database Syst Rev 2006;3:CD004454–4. doi:10.1002/14651858.CD004454.pub2 10.1002/14651858.CD004454.pub2 16856047

[25] Brownfoot FC, Gagliardi DI, Bain E, et al Different corticosteroids and regimens for accelerating fetal lung maturation for women at risk of preterm birth. Cochrane Db Syst Rev 2012;8:CD006764–4. 10.1002/14651858.CD006764.pub3 23990333

[26] Saccone G, Berghella V Antenatal corticosteroids for maturity of term or near term fetuses: systematic review and meta-analysis of randomized controlled trials. BMJ 2016;355:i5044 doi:10.1136/bmj.i5044 27733360 10.1136/bmj.i5044 PMC5062056

[27] Roberts D, Brown J, Medley N, et al Antenatal corticosteroids for accelerating fetal lung maturation for women at risk of preterm birth. Cochrane Database Syst Rev 2017;3:CD004454 doi:10.1002/14651858.CD004454.pub3 28321847 10.1002/14651858.CD004454.pub3 PMC6464568

[28] Vogel JP, Souza JP, Gülmezoglu AM, et al Use of antenatal corticosteroids and tocolytic drugs in preterm births in 29 countries: an analysis of the WHO Multicountry Survey on Maternal and Newborn Health. Lancet 2014;384:1869–77. doi:10.1016/S0140-6736(14)60580-8 25128271 10.1016/S0140-6736(14)60580-8

[29] Fekih M, Chaieb A, Sboui H, et al [Value of prenatal corticotherapy in the prevention of hyaline membrane disease in premature infants. randomized prospective study]. Tunis Med 2002;80:260–5. 12534029

[30] Vogel JP, Souza JP, Gülmezoglu AM, et al Use of antenatal corticosteroids and tocolytic drugs in preterm births in 29 countries: an analysis of the WHO Multicountry Survey on Maternal and Newborn Health. Lancet 2014;384:1869–77. doi:10.1016/S0140-6736(14)60580-8 25128271 10.1016/S0140-6736(14)60580-8

[31] Gyamfi-Bannerman C, Thom EA, Blackwell SC, et al Antenatal Betamethasone for women at risk for Late Preterm delivery. N Engl J Med 2016;374:1311–20. doi:10.1056/NEJMoa1516783 26842679 10.1056/NEJMoa1516783 PMC4823164

[32] Lee K, Khoshnood B, Wall SN, et al Trend in mortality from respiratory distress syndrome in the United States, 1970-1995. J Pediatr 1999;134:434–40. doi:10.1016/S0022-3476(99)70200-3 10190917 10.1016/s0022-3476(99)70200-3

[33] Effect of corticosteroids for fetal maturation on perinatal outcomes. NIH Consensus Development Panel on the effect of corticosteroids for fetal maturation on Perinatal Outcomes. JAMA 1995;273:413–8. 7823388 10.1001/jama.1995.03520290065031

[34] Amorim MM, Santos LC, Faúndes A Corticosteroid therapy for prevention of respiratory distress syndrome in severe preeclampsia. Am J Obstet Gynecol 1999;180:1283–8. doi:10.1016/S0002-9378(99)70630-7 10329891 10.1016/s0002-9378(99)70630-7

[35] Doran TA, Swyer P, MacMurray B, et al Results of a double-blind controlled study on the use of betamethasone in the prevention of respiratory distress syndrome. Am J Obstet Gynecol 1980;136:313–20. doi:10.1016/0002-9378(80)90855-8 7352521 10.1016/0002-9378(80)90855-8

[36] Qublan HS, Malkawi HY, Hiasat MS, et al The effect of antenatal corticosteroid therapy on pregnancies complicated by premature rupture of membranes. Clin Exp Obstet Gynecol 2001;28:183–6. 11530870

[37] Schutte MF, Treffers PE, Koppe JG, et al The influence of betamethasone and orciprenaline on the incidence of respiratory distress syndrome in the newborn after preterm labour. Br J Obstet Gynaecol 1980;87:127–31. doi:10.1111/j.1471-0528.1980.tb04505.x 6988006 10.1111/j.1471-0528.1980.tb04505.x

[38] Lau J, Ioannidis JP, Terrin N, et al The case of the misleading funnel plot. BMJ 2006;333:597–600. doi:10.1136/bmj.333.7568.597 16974018 10.1136/bmj.333.7568.597 PMC1570006

[39] Althabe F, Belizán JM, McClure EM, et al A population-based, multifaceted strategy to implement antenatal corticosteroid treatment versus standard care for the reduction of neonatal mortality due to preterm birth in low-income and middle-income countries: the ACT cluster-randomised trial. Lancet 2015;385:629–39. doi:10.1016/S0140-6736(14)61651-2 25458726 10.1016/S0140-6736(14)61651-2 PMC4420619

[40] McClure EM, Goldenberg RL, Jobe AH, et al Reducing neonatal mortality associated with preterm birth: gaps in knowledge of the impact of antenatal corticosteroids on preterm birth outcomes in low-middle income countries. Reprod Health 2016;13:61 doi:10.1186/s12978-016-0180-6 27221397 10.1186/s12978-016-0180-6 PMC4877818

[41] Vogel JP, Oladapo OT, Manu A, et al New WHO recommendations to improve the outcomes of preterm birth. Lancet Glob Health 2015;3:e589–e590. doi:10.1016/S2214-109X(15)00183-7 26310802 10.1016/S2214-109X(15)00183-7

[42] World Health Organization. WHO recommendations on interventions to improve preterm birth outcomes[Internet]. Geneva: World Health Organization, 2015 http://www.who.int/reproductivehealth/publications/maternal_perinatal_health/preterm-birth-guidelines/en/26447264

